# Retrieval of an infectious leadless pacemaker with vegetation

**DOI:** 10.1002/joa3.12814

**Published:** 2023-01-04

**Authors:** Junji Morita, Yusuke Kondo, Daisuke Hachinohe, Takayuki Kitai, Tsutomu Fujita

**Affiliations:** ^1^ Department of Cardiology Sapporo Cardiovascular Clinic Sapporo Japan; ^2^ Department of Cardiovascular Medicine Chiba University Graduate School of Medicine Chiba Japan

**Keywords:** atrioventricular block, infection, lead extraction, leadless pacemaker, vegetation

## Abstract

This case discusses the retrieval of a pacemaker with vegetation from a 78‐year‐old man. It suggests that grasping side of Micra body and pulling Micra into Agilis sheath is a possible technique for retrieval.

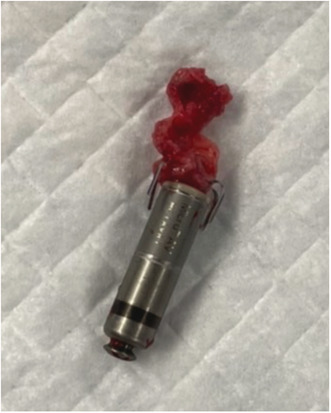

A 78‐year‐old man with bradycardia and fever, diagnosed with coronavirus disease 2019 (COVID‐19) by polymerase chain reaction, and complete atrioventricular (AV) block by electrocardiography, was transferred to our hospital for pacemaker implantation. On admission, cardiac enzyme and ST levels of electrocardiogram were normal.

Echocardiography showed normal left ventricular function and no evidence of vegetation or annulus abscess. Laboratory tests showed a white blood cell count of 7.4 × 103/μl and C‐reactive protein levels of 5.0 mg/dl, and blood culture was negative.

Based on the above, we considered there was not enough evidence to support AV block in this case originated from either COVID‐19‐associated myocarditis or infective endocarditis.

We performed temporary pacing on the first day, and the Micra transcatheter pacing system (Medtronic, Minneapolis, MN, USA) was implanted on the 11th day when the patient's body temperature had returned to normal. The complete AV block was persistent. There were no abnormal findings at the time of the procedure (Figure 1A). The pacing capture threshold was 0.75 V at 0.24 ms, R‐wave amplitude was 6.2 mV, and pacing impedance was 650 Ω. The patient developed a recurrent fever from the day after implantation. C‐reactive protein levels and white blood cell counts were increased. The pacing threshold and impedance were increased (4.5 V at 1.0 ms, 930 Ω) on the fifth day of implantation, and the Micra was lost of capture even at maximum output. Impedance was 1180 Ω on the seventh day; therefore, temporary pacemaker implantation was performed. Chest X‐ray showed no change in Micra location (Figure 1B). Methicillin‐resistant *Staphylococcus aureus* (MRSA) was detected in the blood and antibiotics were administered for 2 weeks; however, the fever did not subside. Transthoracic echocardiography showed no vegetation, but transesophageal echocardiography showed vegetation around the Micra (Figure 2, Video S1). We diagnosed Micra infection and opted to retrieve it. We utilized an Osypka snare catheter (Osypka Medical GmBH, Berlin, Germany) and an 8.5 F steerable sheath (Agilis NXT; St. Jude Medical, St Paul, MN, USA). We failed in catching the retrieval cap using the Osypka snare catheter. When we gripped the retrieval side of the Micra body (Figure 3A), it turned vertically, and we could pull it into an Agilis sheath (Figure 3B–D, and Video S2). Subsequently, vegetation attachment to the Micra was confirmed (Figure 4). Bacterial culture was negative in the vegetation; however, histological examination showed dense neutrophils, which were consistent with the presence of an infection. After retrieval of the Micra, symptoms of infection improved and blood culture became negative. Although we recommended a leadless pacemaker for re‐implantation, the patient preferred a conventional pacemaker. He underwent conventional pacemaker implantation after 4 weeks of continued antibiotics administration and was discharged without recurrence of infection.

**FIGURE 1:**
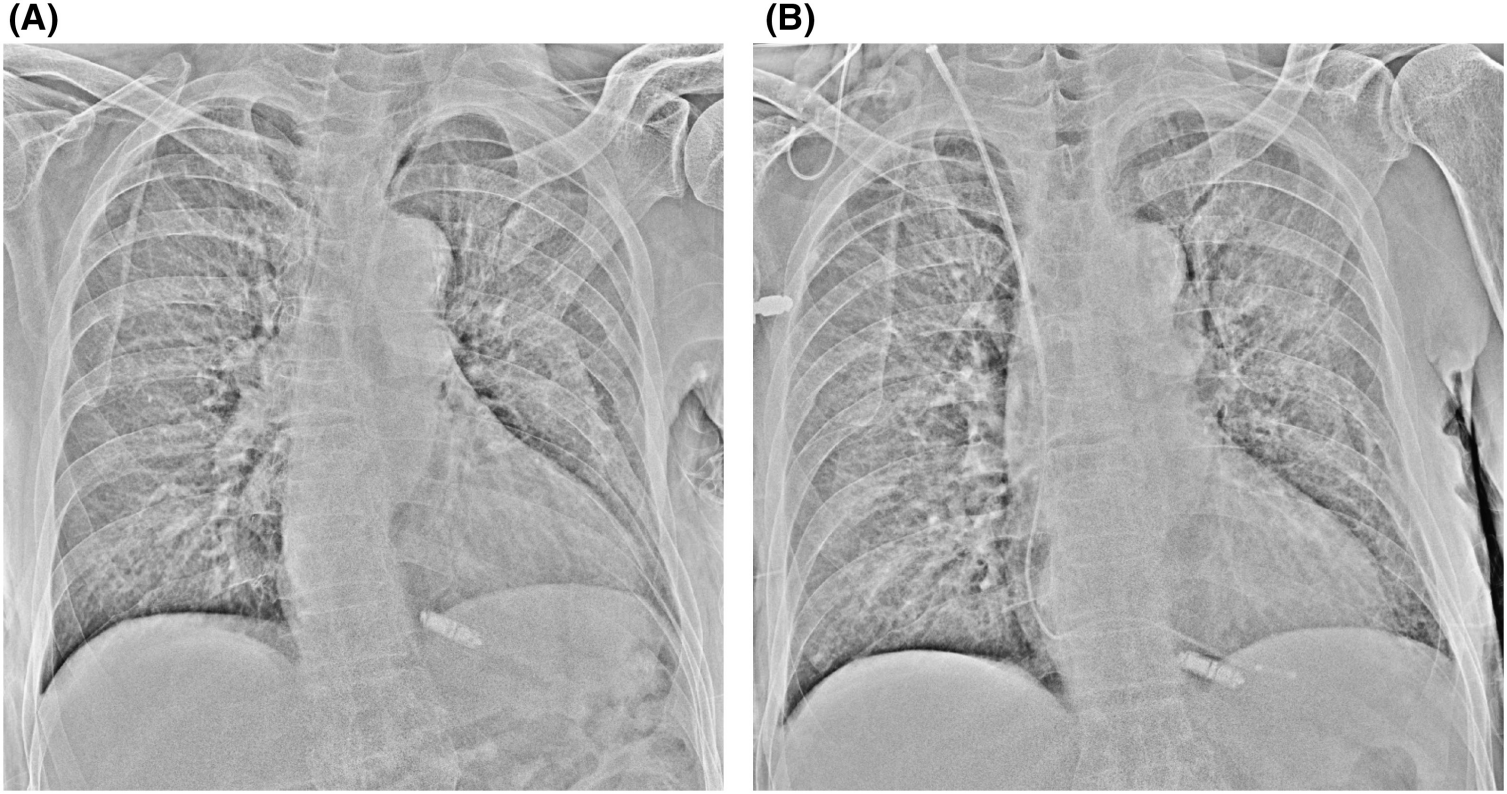
(A) Chest X‐ray immediately after Micra implantation. (B) Chest X‐ray shows no change in Micra location when it could not be captured even at maximum output.

**FIGURE 2:**
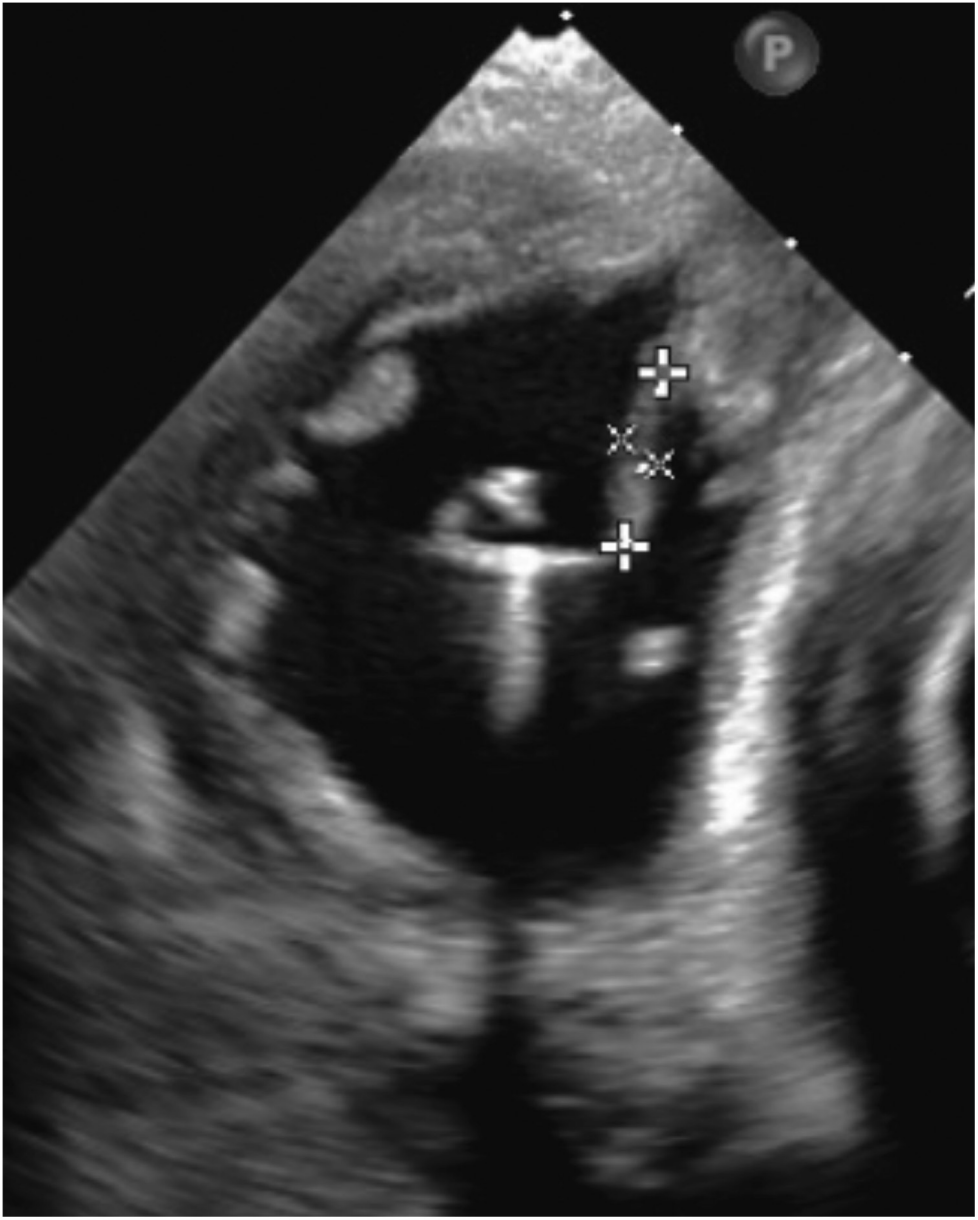
Transesophageal echocardiography showing vegetation.

**FIGURE 3:**
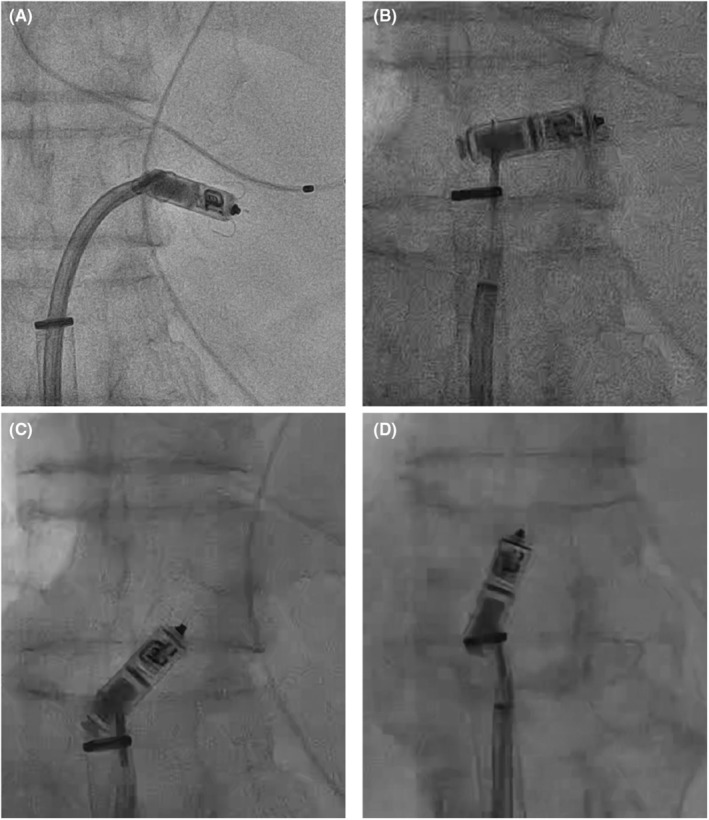
(A and B) Grabbing and pulling the Micra body. (C) Turning the Micra vertically. (D) Pulling the Micra into the Agilis sheath.

**FIGURE 4:**
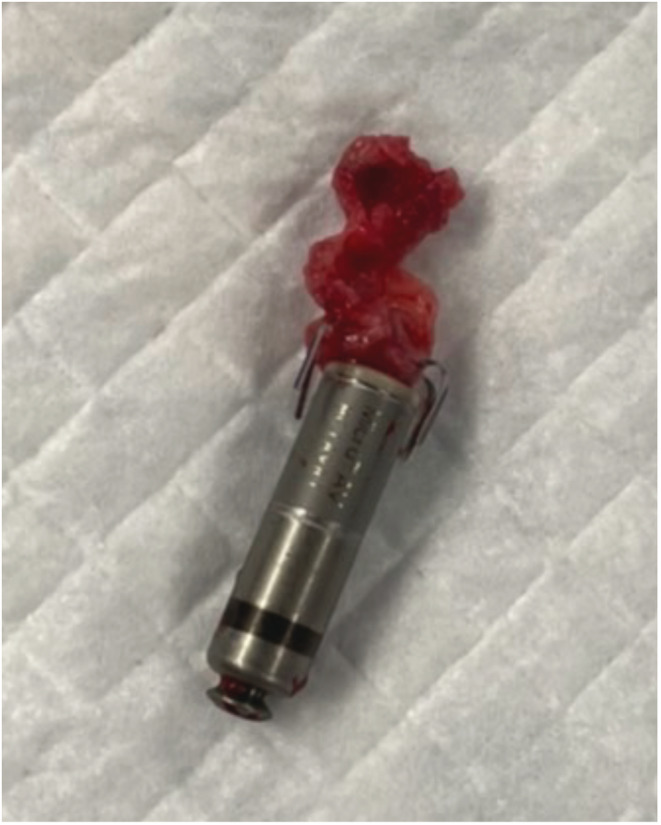
Micra with vegetation.

Leadless pacemakers are generally considered less susceptible to infections. No postoperative infection was found in the Investigational Device Exemption study.^1^ Some studies report that simultaneous leadless pacemaker implantation and CIED extraction are feasible even in active infections.^2^ Conversely, there are several reports of infection from leadless pacemakers.^3^, ^4^ In these cases, vegetation was attached to the leadless pacemaker after implantation, and MRSA sepsis was observed. Temporary pacing has been identified as a risk factor for pacemaker infections. Temporary pacing and immunodeficiency after COVID‐19 infection may have caused infection of the Micra transcatheter. Regarding patients with a high risk of infection, leadless pacemakers may require careful management similar to conventional pacemakers. The pacing threshold and impedance increased gradually, and finally, loss of capture occurred even at maximum output. The vegetation attachment to the Micra electrodes may increase pacing impedance and block the electric current, while additionally, inflammation of the myocardium may affect pacing thresholds.

When fever and pacing threshold exacerbation are observed after Micra implantation, a device infection should be suspected.

Various studies have reported Micra removal, and the method of removal was generally gripping the retrieval cap. However, the retrieval cap of Micra is too small to capture with a snare. Hence, alternative techniques have been reported for Micra removal, including one case where it was removed using two snares to grip the retrieval cap.^5^ To the best of our knowledge, this is the first case wherein the Micra body was grasped and pulled inside the sheath. It is possible to grasp the retrieval side of the body with the Osypka snare catheter with a strong gripping force. The technique of grasping the Micra body and not the retrieval cap is simple. Moreover, this method is considered useful when the retrieval cap is wrapped in an organization of vegetation and surrounding tissue. On the other hand, we propose that grabbing the Micra cap is preferable in terms of coaxiality. We suggest grabbing the Micra body instead of the retrieval cap as an alternative method, when capturing of the latter is difficult.

## CONFLICT OF INTEREST

Dr. Kondo received lecture fees from Daiichi‐Sankyo, Bayer, Abbott Medical Japan, Biotronik Japan, Boston Scientific, and Japan Lifeline, and research funds from Daiichi‐Sankyo. The other authors have no conflicts of interest to declare.

## ETHICS STATEMENT

This study was conducted according to the principles of the Declaration of Helsinki. The study was approved by the Institutional Review Board.

## PATIENT CONSENT STATEMENT

The patient provided written informed consent.

## Supporting information


Video S1.

Video S2.

VideoCaptions

## References

[1] Reynolds D , Duray GZ , Omar R , Soejima K , Neuzil P , Zhang S , et al. A leadless intracardiac transcatheter pacing system. NEngl J Med. 2016;374:533–41. 10.1056/NEJMoa1511643 26551877

[2] Chang D , Gabriels JK , Kim BS , Ismail H , Willner J , Beldner SJ , et al. Concomitant leadless pacemaker implantation and lead extraction during an active infection. J Cardiovasc Electrophysiol. 2020;31:860–7. 32048776 10.1111/jce.14390

[3] Koay A , Khelae S , Wei KK , Muhammad Z , Ali RM , Omar R . Treating an infected transcatheter pacemaker system via percutaneous extraction. HeartRhythm Case Rep. 2016;2:360–2. 28491710 10.1016/j.hrcr.2016.04.006 PMC5419892

[4] Nozoe M , Yoshida D , Nagatomo D , Suematsu N , Kubota T , Okabe M , et al. Successful percutaneous retrieval of a micra transcatheter pacing system at 8 weeks after implantation. J Arrhythm. 2018;34:653–5. 30555612 10.1002/joa3.12119 PMC6288601

[5] Hasegawa‐Tamba S , Ikeda Y , Tsutsui K , Kato R , Muramatsu T , Matsumoto K . Two‐directional snare technique to rescue detaching leadless pacemaker. HeartRhythm Case Rep. 2020;10:711–4. 10.1016/j.hrcr.2020.06.027 PMC7573372 33101938

