# Supplementary material for: Retrieval of an infectious leadless pacemaker with vegetation
Source: J Arrhythm. 2023 Jan 4;39(1):71–3. doi: 10.1002/joa3.12814 (PMC9885316; doi:10.1002/joa3.12814)
Supplement: Supplementary file 3 — VideoCaptions [file JOA3-39-71-s001.docx]

Video S1. Transesophageal echocardiography showing vegetation

Video S2. Retrieval of the Micra
